# Genes of the Glutamatergic System and Tardive Dyskinesia in Patients with Schizophrenia

**DOI:** 10.3390/diagnostics12071521

**Published:** 2022-06-22

**Authors:** Olga Yu. Fedorenko, Diana Z. Paderina, Elena G. Kornetova, Evgeniya G. Poltavskaya, Ivan V. Pozhidaev, Anastasiia A. Goncharova, Maxim B. Freidin, Anna V. Bocharova, Nikolay A. Bokhan, Anton J. M. Loonen, Svetlana A. Ivanova

**Affiliations:** 1 Mental Health Research Institute, Tomsk National Research Medical Center of the Russian Academy of Sciences, 634014 Tomsk, Russia; f_o_y@mail.ru (O.Y.F.); osmanovadiana@mail.ru (D.Z.P.); kornetova@sibmail.com (E.G.K.); egboyarko@mail.ru (E.G.P.); craig1408@yandex.ru (I.V.P.); goncharanastasya@gmail.com (A.A.G.); nikolay.bokhan.tomsk.russia@gmail.com (N.A.B.); ivanovaniipz@gmail.com (S.A.I.); 2 Department of Psychiatry, Addictology and Psychotherapy, Siberian State Medical University, 634050 Tomsk, Russia; 3 Research Institute of Medical Genetics, Tomsk National Research Medical Center of the Russian Academy of Sciences, 634050 Tomsk, Russia; m.freydin@qmul.ac.uk (M.B.F.); anna.bocharova@medgenetics.ru (A.V.B.); 4 School of Biological and Behavioural Sciences, Queen Mary University of London, London E1 4NS, UK; 5 Unit of PharmacoTherapy, Epidemiology & Economics, Groningen Research Institute of Pharmacy, University of Groningen, 9713 AV Groningen, The Netherlands

**Keywords:** schizophrenia, antipsychotics, tardive dyskinesia, glutamatergic system, genes, pharmacogenetics, microglia

## Abstract

Background: Tardive dyskinesia (TD) is an extrapyramidal side effect of the long-term use of antipsychotics. In the present study, the role of glutamatergic system genes in the pathogenesis of total TD, as well as two phenotypic forms, orofacial TD and limb-truncal TD, was studied. Methods: A set of 46 SNPs of the glutamatergic system genes (*GRIN2A*, *GRIN2B*, *GRIK4*, *GRM3*, *GRM7*, *GRM8*, *SLC1A2*, *SLC1A3*, *SLC17A7*) was studied in a population of 704 Caucasian patients with schizophrenia. Genotyping was performed using the MassARRAY Analyzer 4 (Agena Bioscience™). Logistic regression analysis was performed to test for the association of TD with the SNPs while adjusting for confounders. Results: No statistically significant associations between the SNPs and TD were found after adjusting for multiple testing. Since three SNPs of the *SLC1A2* gene demonstrated nominally significant associations, we carried out a haplotype analysis for these SNPs. This analysis identified a risk haplotype for TD comprising CAT alleles of the *SLC1A2* gene SNPs rs1042113, rs10768121, and rs12361171. Nominally significant associations were identified for *SLC1A3* rs2229894 and orofacial TD, as well as for *GRIN2A* rs7192557 and limb-truncal TD. Conclusions: Genes encoding for mGlu3, EAAT2, and EAAT1 may be involved in the development of TD in schizophrenia patients.

## 1. Introduction

Schizophrenia is one of the most severe polymorphic mental disorders and is characterized by positive and negative symptoms, as well as behavioral and cognitive impairments [1,2,3]. According to a systematic review of 129 individual data sources, the global age-standardized point prevalence of schizophrenia in 2016 was estimated to be 0.28% (95% uncertainty interval (UI): 0.24–0.31) [4]. The main components of the genesis of schizophrenia are a combination of genetic predisposition factors and exposure to adverse environmental factors [5]. Schizophrenia is a highly polygenic disorder with a complex array of risk loci [6,7,8].

The treatment of schizophrenia still relies primarily on patients having to use antipsychotics. However, due to long-term treatment with typical and atypical antipsychotics, various adverse events may occur, such as neuroendocrine, metabolic, and extrapyramidal side effects [9,10,11,12]. Tardive dyskinesia is a severe motor side effect of antipsychotic drugs characterized by involuntary rapid muscle contractions and athetoid movements of the trunk, limbs, and orofacial muscles [13,14,15]. The exact mechanism of TD is still unknown, but we previously presented evidence that it could be related to the impairment of the GABAergic medium spiny projection neurons (MSNs) of the indirect extrapyramidal pathway [16]. In the establishment of this damage, oxidative stress, due to intracellular excess dopamine (due to a dopamine D2 receptor blockade) and glutamatergic excitotoxicity, may play a mechanistic role [16]. In addition to dopamine receptors, serotonin receptors can also be associated with the occurrence of TD [17]. Already in one of the first studies of our group, we found evidence that different genetic factors contribute to the development of orofacial/classical TD in comparison to limb-truncal/peripheral dyskinesia [18]. This may be related to the fact that orofacial movements are associated with phylogenetically older brain structures than those of the limbs [19,20]. Motor side effects of antipsychotic therapy remain understudied despite their prevalence [21].

Glutamate is the major excitatory amino acid neurotransmitter in the mammalian central nervous system [22]. It plays an important role in numerous physiological functions, including learning and memory, sensory perception, the development of synaptic plasticity, motor control, respiration, and the regulation of cardiovascular function. Glutamate realizes its action through two types of glutamate receptors; ionotropic receptors mediate fast synaptic transmission and metabotropic G-protein coupled receptors mediate slow synaptic transmission [23]. Both types of receptors are associated with the development of TD [24]. However, the possible association between the genetic variants of the genes encoding (parts of) these receptors were studied almost exclusively for the NR2A and NR2B subunits of the ionotropic glutamate N-methyl-D-aspartate (NMDA) receptor [19].

Ionotropic glutamate receptors can be divided into three families, of which several subtypes exist: α-amino-3-hydroxy-5-methyl-4-isoxazolepropionic acid (AMPA), kainate, and N-methyl-D-aspartate (NMDA) receptors [25,26,27]. These subunits form tetramers with each other, which act as ion channels that depolarize the membrane. Thus, the NMDA ion channels are also permeable to Ca^2+^ ions and therefore capable of the long-term potentiation of synaptic excitation.

The tetrameric NMDA receptor is composed of subunits, which exist in different forms: GluN1 (product of *GRIN1*), GluN2 (*GRIN2A*, *GRIN2B*, *GRIN2C*, and *GRIN2D*), and GluN3 (*GRIN3A* and *GRIN3B*) [25,26,27]. Most NMDA receptors consist of two GluN1 subunits (to which glycine can bind) and two GluN2 subunits (to which glutamate can). *GRIN2A* is located on chromosome 16p13.2 and codes for a polypeptide of 1464 amino acid residues, GluN2A (also called NR2A) [25]. *GRIN2B* is located on chromosome 12p12 and codes for a polypeptide of 1484 amino acid residues, GluN2B or NR2B [25]. These genes have been investigated for possible involvement in the pathophysiology of TD in patients with schizophrenia, but the results of the studies are inconsistent [28,29,30].

The kainate receptor is also composed of four peptide molecules, of which a total of five different variants exist, named GluK1 (or GRIK1) to GluK5 (or GRIK5). The *GRIK4* gene encoding the protein product KA1, 956 amino acid residues in size, is located on chromosome 11q22.3. To our knowledge, the possible existence of an association of genetic variants of this gene was investigated for the pathophysiology of schizophrenia [31] but not for TD.

The metabotropic glutamate (mGlu) receptors include eight different G-protein coupled receptors, which are divided into three groups based on the homology of their amino acid sequences: Group I (mGluR1 and mGluR5), Group II (mGluR2 and mGluR3), and Group III (mGluR4, mGluR6, mGluR7, and mGluR8) [32,33]. mGluR3, mGluR7, and mGluR8 are linked to a Gi/o protein for signal transducing. The *GRM3*, *GRM7*, and *GRM8* genes encoding the metabotropic glutamate receptors mGluR3, mGluR7, and mGluR8, respectively, are located on chromosomes 7q21.11-q21.12, 3p26.1, and 7q31.33-q32.1, respectively. They have been studied for a possible association with schizophrenia [34,35,36], but we found no pharmacogenetic studies on the possible role of these genes in the development of extrapyramidal side effects [19].

To function as a neurotransmitter, glutamate must be removed from the synaptic cleft after its release by (re)uptake by surrounding neurons and glia cells and then stored in presynaptic storage vesicles or metabolized. The former is done by excitatory amino acid transporters (EAATs), of which 5 variant polypeptides exist in humans in the range of 500–600 amino acid residues; EAAT1 to EAAT5 [37,38]. The *SLC1A2* gene is localized on chromosome 11p13 and encodes for the brain’s main glutamate transporter; the excitatory amino acid transporter 2 (EAAT2). Recently, an association of a genetic variant of this gene with schizophrenia was found [39], and a relationship between EAAT2 functioning and schizophrenia and mood disorders was also previously suggested [40]. The *SLC1A3* encodes for EAAT1 and is localized in chromosome 5p13.2. Mutations of this gene are thought to be responsible for the development of certain forms of episodic ataxias [41,42]. Three subtypes of the vesicular glutamate transporter exist of approximately 600 amino acid residues in size (VGLUTs 1, 2, and 3) [38]. The *SLC17A7* gene on chromosome 19q13.33 encodes the VGLUT1 protein, which, in humans, consists of 560 amino acids [43]. VGLUT1 is mainly expressed by glutamatergic terminals in the cerebral cortex, hippocampus, and basolateral amygdala [44].

The present study aimed to investigate the role of polymorphic variants of the genes for ionotropic receptors (*GRIN2A*, *GRIN2B*, *GRIK4*), metabotropic receptors (*GRM3*, *GRM7*, *GRM8*), and glutamate transporters (*SLC1A2*, *SLC1A3*, *SLC17A7*) in the development of tardive dyskinesia and its subtypes in patients with schizophrenia.

## 2. Materials and Methods

### 2.1. Patients

The study was conducted in accordance with the Code of Ethics of the World Medical Association (Declaration of Helsinki 1975, as revised in Fortaleza, Brazil, 2013). The protocol of this study was submitted to and approved by the Bioethics Committee of the Mental Health Research Institute of the Tomsk National Research Medical Center of the Russian Academy of Sciences (protocol No. 142, approved on 14 May 2021). The study group consisted of a total of 944 patients with schizophrenia treated at the clinics of the Mental Health Research Institute of the Tomsk National Research Medical Center, the Tomsk Clinical Psychiatric Hospital, and the Kemerovo Regional Clinical Psychiatric Hospital. Informed consent was obtained from all subjects involved in the study.

The inclusion criteria for the study were a verified clinical diagnosis of schizophrenia in accordance with the International Classification of Diseases, 10th revision (ICD-10) criteria [45], age 18–65 years, the absence of organic, certain neurological, and severe somatic pathology, and the presence of a signed informed consent form for the study. The examined patients are residents of the Siberian Region, of White clinical appearance, and not related by blood to each other.

The exclusion criteria included the presence of pharmacological withdrawal symptoms, as well as comorbid neurological, organic, and somatic diseases that would make it difficult to objectively assess the clinical condition due to the underlying disease.

The patients studied had been taking antipsychotic treatment with typical and/or atypical antipsychotics for a long time. In the study, chlorpromazine equivalent (CPZeq) was used to standardize the dose, efficacy, and side effects of antipsychotics [46]. To determine the presence and severity of dyskinesia, patients were examined with the Abnormal Involuntary Movement Scale (AIMS) [13,14,15,47].

### 2.2. Genetic Analysis

Antecubital venous blood for research was taken into BD Vacutainer tubes with EDTA anticoagulant. DNA extraction was carried out by the standard phenol-chloroform method. DNA concentration measurements were performed on a Thermo Scientific NanoDrop 8000 UV–Vis Spectrophotometer.

Genotyping of 46 single nucleotide polymorphisms of the genes of the glutamatergic system was performed: *GRIN2A* (rs11644461, rs11646587, rs11866328, rs7190619, rs7192557, rs7196095, rs7206256, rs9788936, rs9989388, rs1345423, rs4782039, rs8057394), *GRIN2B* (rs10772715, rs10845838, rs12300851, rs12827536, rs1805481, rs220599, rs2300242, rs7313149, rs2192970, rs890), *GRIK4* (rs1954787), *GRM3* (rs1468412, rs2237562, rs6465084, rs2299225), *GRM7* (rs1396409, rs3749380, rs12491620, rs1450099, rs17031835), *GRM8* (rs2237748, rs2299472), *SLC1A2* (rs1042113, rs10768121, rs12361171, rs3829280, rs10742338, rs11033046, rs12294045, rs3088168, rs3812778, rs7936950, *SLC1A3* (rs2229894), and *SLC17A7* (rs62126236). SNPs that were mentioned in the previous genetic studies mentioned in the introduction and had a minor allele frequency (MAF) of >5% were studied. The basic information on these SNPs is described in Table 1.

Genotyping was performed on a mass spectrometer MassARRAY^®^ Analyzer 4 (Agena Bioscience™, San Diego, CA, USA) using the set Consumables iPLEX Gold 96 on The Core Facility “Medical Genomics” base, Tomsk NRMC.

### 2.3. Statistical Analysis

Statistical analysis was performed in the R 4.0.4 software environment using basic functions and the “haplo.stats” package. The Hardy–Weinberg equilibrium (HWE) of genotypic frequencies was tested using the χ2 test. Logistic regression was applied to test the association between tardive dyskinesia and genetic variants (additive model) while correcting for age and sex. Bonferroni correction was applied after calculating the number of independent tests, following the approach described by Li and Li [48] and after excluding the SNPs that were different from the HWE. Associations were considered statistically significant at *p* < 0.05 after correction for multiple testing.

## 3. Results

A total of 944 patients (435 females and 509 males) receiving long-term antipsychotic therapy were examined. Table 2 presents the main demographic and clinical parameters of the studied patient groups.

Tardive dyskinesia was diagnosed in 229 patients (24.3%). The median age of patients with tardive dyskinesia was statistically significantly higher than in the comparison group (*p* < 0.001). As not unexpected in relation to this, the duration of illness was also longer in the group of patients with tardive dyskinesia (*p* < 0.001).

Out of the total group of 944 patients with schizophrenia, we genotyped a smaller representative sample of 704 patients (Table 3).

An analysis of the allelic variants of the studied genes showed that the observed distribution of the genotype frequencies corresponded to that expected at the Hardy–Weinberg equilibrium. The exceptions were two polymorphic variants, *SLC1A2* rs10742338 and *GRM3* rs6465084, which were excluded from the following statistical analysis.

A total of 33 independent SNPs were estimated following the procedure described in [48]. Thus, for the analysis of associations, the significance level after Bonferroni correction was set as 0.05/33 = 0.0015.

No statistically significant associations between the SNPs and TD were found after adjusting for multiple testing (Table 4).

It has been noted that three SNPs of the *SLC1A2* gene demonstrated nominally significant associations; therefore, we carried out haplotype analysis for these SNPs. This analysis identified a risk haplotype for TD comprising the CAT alleles of the *SLC1A2* gene SNPs rs1042113, rs10768121, and rs12361171 (Table 5).

The same statistical analysis was also performed on subgroups of patients with various phenotypic forms of tardive dyskinesia: orofacial and limb-truncal. Nominally significant associations were identified for rs2229894 of *SLC1A3* and orofacial TD (β = −0.4566 ± 0.1855, *p* = 0.0138) as well as for rs7192557 of *GRIN2A* and limb-truncal TD (β = 0.4385 ± 0.1958, *p* = 0.0251).

## 4. Discussion

In this article, we present the results of a study on the possible association of 44 polymorphisms of genes involved in glutamatergic neurotransmission with the prevalence of classical, peripheral, and total tardive dyskinesia in 704 out of 944 patients with schizophrenia from clinics in Western Siberia in the Russian Federation. For six SNPs (*GRM3* rs2237562 and rs1468412; *SLC1A2* rs1042113, rs10768121, and rs12361171; and *SLC1A3* rs2229894), we found a nominally significant association with TD, but none of them survived Bonferroni correction for multiple testing. Haplotype analysis for *SCL1A2* rs1042113, rs10768121, and rs12361171 identified a risk haplotype for TD comprising CAT alleles. In addition, logistic regression analysis revealed nominally significant associations for *SLC1A3* rs2229894 and orofacial TD, as well as for *GRIN2A* rs7192557 and limb-truncal TD.

Glutamate is the main excitatory neurotransmitter of the central nervous system, and its neurons are widely distributed in the brain. Therefore, the dysfunction of this system can lead to a wide range of neuropsychiatric symptoms. Excitatory transmission is achieved through the activation of ion-channel or G protein-coupled glutamate receptors. The intensity of receptor interaction is determined in part by the removal of glutamate from the synaptic cleft and by neurotransmitter storage in the presynaptic vesicles. In addition to direct neurotransmission, glutamate is also important for inducing neuroplastic changes and causing excitotoxicity [49]. Within the extrapyramidal system, striatal MSNs, among others, are innervated by glutamatergic corticostriatal and thalamostriatal neurons [19]. Glutamatergic neurons from all parts of the cerebral cortex terminate in ‘striatal spine module’ of matrix MSNs of the extrapyramidal cortical–striatal–thalamic–cortical (CSTC) circuits, while projection neurons from the prefrontal cortex and corticoid amygdala terminate in the striosomal compartment of the striatum [19]. The latter neurons finally regulate the activity of the ascending dopaminergic pathway systems originating within the midbrain. Glutamatergic corticostriatal neurons targeting both compartments may play a role in the development of tardive dyskinesia. Excessive glutamatergic activity in striatal spine modules may result in damage to the MSNs of the indirect pathway. This may also be due to an excessive release of dopamine from mesostriatal neurons, which increases oxidative stress in the MSNs of the indirect pathway along that pathway. The MSNs of the indirect pathway are more susceptible to damage than those of the direct pathway [16,19].

We already investigated the possible association of polymorphisms of *GRIN2A* and *GRIN2B* in a previous study in a smaller patient population [29] and compared our findings with those of 168 Dutch White patients by Bakker and others [50]. The results hardly agreed. Only for rs1345423 in *GRIN2A* was a significant association with TD found in both populations. The *GRIN2A* variants rs7192557 (rs1969060) and rs8057394 were associated with the age of onset of dyskinesia in Huntington’s disease [51,52,53]. Ivanova et al. observed a relationship between these genetic variants and levodopa-induced dyskinesia but not with TD [28]. The various findings are confirmed in the presently studied patient population, suggesting that the NMDA receptor plays, at most, a minor role in the development of TD. However, it is at least notable that the polymorphism rs7192557, which has been associated with the onset of dyskinesia in Huntington’s disease [51,52,53] as well as with levodopa-induced dyskinesia [28], shows some association with peripheral TD in the present study.

Neuroplasticity and excitotoxicity are usually associated mainly with the properties of NMDA receptors, but the kainate receptor is, at least theoretically, also a good candidate. Kainic acid is best known for its excitotoxic effect [54,55] and is used in a classical model for HD [56,57] and epilepsy [58,59]. A certain *GRIK2* variant was associated with the age of onset of HD, but this has been later contradicted [52,60]. The GRIK4 kainate receptor was strongly associated with excitotoxic neurodegeneration (in the hippocampus) [61], but nothing is known about its relation to movement disorders. Our results also do not point in the direction of a possible link.

In addition to the ionotropic glutamate receptors, the metabotropic (mGlu) receptors are also associated with processes of neurodegeneration/neuroprotection [62,63]. Because the various mGlu receptors are also localized in the extrapyramidal system [63,64,65], the value of drugs with an affinity for mGlu receptors was also investigated in Parkinson’s disease [65]. This led to the development of many new drugs that may be of value in the prevention and treatment of LID [65]. Unfortunately, the clinical results of substances targeting mGlu4 and mGlu5 were rather disappointing [66]. In our study, we found a nominally significant influence of variants of mGlu3. Apart from the axons of neurons, mGlu3 receptors are also found on reactive astrocytes [64] and microglia [67]. Through these receptors, mGlu3 receptors mediate the bringing of microglia into an anti-inflammatory state, which has a significant protective effect against neurodegenerative processes [68].

The vesicular glutamate transporter (VGLUT) plays a role in concentrating glutamate in its presynaptic vesicles, which is a prerequisite for glutamatergic neurotransmission. Of the three forms that exist of this transporter, VGLUT1 and VGLUT2 are the most abundant but complementary in the central nervous system [38,69]. VGLUT1 is used as a biomarker for corticostriatal glutamatergic neurons, whereas VGLUT2 is mainly present in thalamostriatal glutamatergic neurons [70]. The involvement of VGLUT1 in the pathogenesis of CNS disorders has not actually been investigated very thoroughly [71]. There is some evidence for a possible role in Parkinson’s disease and schizophrenia, but this appears to be primarily cognitive in nature [71].

The solute carrier family 1 member 2 (*SLC1A2*) gene codes for the excitatory amino acid transporter 2 (EAAT2) protein, and the solute carrier family 1 member 3 (*SLC1A3*) for the EAAT1 protein. Excitatory amino acid transporter 1 is mainly found in glia cells in the cerebellum but is also present in glia cells throughout the CNS [72]. EAAT2, which is also predominantly present in glial cells, is widespread and abundant in the forebrain, cerebellum, and spinal cord, and accounts for more than 90% of the total glutamate intake [38,72]. Both astrocytes [73,74] and (activated) microglia [75,76,77,78] carry EAAT1 and EAAT2 proteins and, to a much lesser extent, neurons [72,79]. Significance was attributed to EAAT1 and EAAT2 in the development of symptoms of various mental illnesses [79]. We also consider it significant that astrocytes are an important part of the striatal spinal modules [19] and regulate extracellular glutamate concentrations by their uptake [38, 40]. However, it is somewhat illogical to assume that this mechanism contributes to the development of (classical) TD, as interference with glutamatergic neurotransmission appears to have little effect, as reflected by the absence of other associations. One might also consider a mechanism influencing neurotransmission in the habenular complex, where astrocytes and the immunocompetent microglia also play an important regulatory role [79].

### Limitations

Although our genotyped population counted a substantial 704 patients, only 158 people (28.9%) had TD. We used a good reliable variant of the AIMS to evaluate the presence of TD, but a limitation remains with the one-time cross-sectional assessment. Patients with TD were significantly older and sicker than individuals without TD, so the exposure to antipsychotics probably lasted longer. This may mean that the group without TD probably also includes individuals who would have exhibited TD on longer exposure. We do not have sufficient information about this medication history and have to accept that this may have resulted in a bias. However, it is unlikely that this bias is very large because the interference of antipsychotics with glutamatergic neurotransmission is, at best, modest and usually absent.

## 5. Conclusions

The results of our study point to the involvement of excitatory amino acid transporter 1 (EAAT1) and 2 (EAAT2), as well as metabotropic glutamate receptor 3 (mGlu3) in the development of TD. The possibility should be considered and explored that this is primarily via the role of glutamate in glial cell function (particularly considering neuroglia in the habenula). The possible role of microglia in the development of TD warrants further investigation. Hence, glutamate plays a role in the development of TD but probably not through glutamatergic neuronal fibers (as, for example, corticostriatal and thalamostriatal inputs to striatal MSNs). The possibility should be considered and explored that this is primarily via the role of glutamate in (neuro)glial cell function.

## Figures and Tables

**Table 1:** The basic information of analyzed polymorphic variants.

Gene	SNP	Chromosome: Location	Location Region	Alleles	MAF	χ^2^	*p*-Value
*GRIN2A*	rs11644461	16:10027033	intron variant	T/C	0.23 (C)	0.686	0.408
	rs11646587	16:9779462	intron variant	G/A	0.29 (A)	0.373	0.541
	rs11866328	16:9768699	intron variant	G/T	0.31 (T)	0	1
	rs1345423	16:10154207	intron variant	G/A/C/T	0.32 (G)	0.241	0.624
	rs4782039	16:9913110	intron variant	T/C	0.22 (C)	0.659	0.417
	rs7190619	16:9985267	intron variant	G/A	0.07 (A)	0.665	0.415
	rs7192557	16:10029612	intron variant	G/A/C/T	0.32 (A)	0.280	0.597
	rs7196095	16:9791975	intron variant	T/C/G	0.30 (C)	0.110	0.740
	rs7206256	16:10103066	intron variant	A/G/T	0.44 (G)	0.417	0.518
	rs8057394	16:10021631	intron variant	C/G	0.43 (C)	0.354	0.552
	rs9788936	16:10011603	intron variant	T/C	0.24 (C)	0.014	0.906
	rs9989388	16:9872282	intron variant	C/T	0.19 (T)	0.255	0.614
*GRIN2B*	rs10772715	12:13885069	intron variant	G/A	0.43 (A)	0.006	0.938
	rs10845838	12:13741462	intron variant	G/A	0.38 (A)	0.482	0.487
	rs12300851	12:13815471	intron variant	T/A/C	0.10 (C)	0.066	0.798
	rs12827536	12:13943223	intron variant	C/T	0.22 (T)	0.912	0.340
	rs1805481	12:13610521	intron variant	A/C	0.43 (C)	0.832	0.362
	rs2192970	12:13683379	intron variant	G/A	0.11 (A)	0.195	0.659
	rs220599	12:13822364	intron variant	G/A	0.44 (A)	2.664	0.103
	rs2300242	12:13687363	intron variant	A/T	0.48 (T)	0.604	0.437
	rs7313149	12:13675353	intron variant	T/A/C/G	0.21 (C)	1.875	0.171
	rs890	12:13562374	3 prime UTR variant	A/C/G	0.28 (C)	0.600	0.438
*GRIK4*	rs1954787	11:120792654	intron variant	T/C	0.50 (T)	0.052	0.819
*SLC1A2*	rs1042113	11:35286822	synonymous variant	T/C	0.23 (C)	0.081	0.777
	rs10742338	11:35255541	3 prime UTR variant	T/A/C	0.10 (T)	4.312	0.038 *
	rs10768121	11:35258109	3 prime UTR variant	A/C/G	0.35 (C)	1.411	0.235
	rs11033046	11:35253386	3 prime UTR variant	T/A	0.36 (A)	0.763	0.382
	rs12294045	11:35257754	3 prime UTR variant	C/G/T	0.20 (T)	0.015	0.904
	rs12361171	11:35256786	3 prime UTR variant	T/A/C	0.36 (A)	2.575	0.109
	rs3088168	11:35251721	3 prime UTR variant	T/C	0.36 (C)	0.778	0.378
	rs3812778	11:35255723	3 prime UTR variant	G/A	0.10 (A)	2.547	0.110
	rs3829280	11:35255176	3 prime UTR variant	A/C/T	0.13 (T)	2.916	0.088
	rs7936950	11:35257412	3 prime UTR variant	C/A/G/T	0.10 (C)	3.488	0.062
*SLC1A3*	rs2229894	5:36686302	3 prime UTR variant	G/A/C	0.43	0.552	0.457
*SLC17A7*	rs62126236	19:49441696	intron variant	T/C	0.19 (C)	0.458	0.499
*GRM3*	rs1468412	7:86804135	intron variant	A/T	0.38 (T)	0	1
	rs2237562	7:86792916	intron variant	T/C	0.39 (C)	0.587	0.444
	rs2299225	7:86818264	intron variant	T/G	0.03 (G)	1.247	0.264
	rs6465084	7:86774159	intron variant	A/G	0.23 (G)	4.100	0.043 *
*GRM7*	rs12491620	3:7352646	intron variant	C/G	0.18 (G)	3.215	0.073
	rs1396409	3:7261220	intron variant	G/A/C/T	0.34 (A)	0.096	0.757
	rs1450099	3:7496689	intron variant	T/G	0.38 (T)	0.099	0.752
	rs17031835	3:6880071	intron variant	C/T	0.10 (T)	0.039	0.844
	rs3749380	3:6861610	missense variant	C/G/T	0.42 (T)	0.299	0.585
*GRM8*	rs2237748	7:126638809	intron variant	C/T	0.32 (T)	0	1
	rs2299472	7:126580415	intron variant	C/A/G	0.32 (A)	0.062	0.803

Notes: MAF—minor allele frequency; HWE χ2 and HWE *p*—chi-square and *p*-value statistics, respectively, to test the frequency distribution according to the Hardy–Weinberg equilibrium. * and in bold: significant at *p* < 0.05.

**Table 2:** Demographic and clinical parameters of the total group of patients with schizophrenia depending on the presence or absence of tardive dyskinesia.

	Patients without TD	Patients with TD	*p*-Value
Total sample size	715	229	-
Gender, n (%)	Male—375 (52.45%)	Male—134 (58.51%)	0.109
	Female—340 (47.55%)	Female—95 (41.49%)	
Age, years Me (Q1; Q3)	37 (30; 48)	45 (34.75; 56.25)	<0.001
Age of onset, years Me (Q1; Q3)	24 (20; 29)	24 (19; 31.25)	0.558
Duration of illness, years Me (Q1; Q3)	11.5 (5; 20)	18 (8.75; 27.25)	<0.001
CPZeq, dose Me (Q1; Q3)	450 (225; 750)	500 (300; 758.7)	0.187

Notes: Me (Q1; Q3)—median and quartiles (first and third); TD—tardive dyskinesia; CPZeq—chlorpromazine equivalent (according to [46]).

**Table 3:** Demographic and clinical parameters of the representative group of patients genotyped.

	Patients without TD	Patients with TD	*p*-Value
Total sample size	546	158	-
		Orofacial TD—86 Limb-truncal TD—77	
Gender, n (%)	Male—282 (51.65%)	Male—88 (55.70%)	0.109
	Female—264 (48.35%)	Female—70 (44.30%)	
Age, years Me (Q1; Q3)	38 (31; 48)	45 (34; 57)	<0.001
Age of onset, years Me (Q1; Q3)	24 (20; 30)	24 (19.5; 31.5)	0.558
Duration of illness, years Me (Q1; Q3)	12 (6; 21)	18 (9; 27)	<0.001
CPZeq, dose Me (Q1; Q3)	430 (225; 779.95)	500 (300; 758.7)	0.294

Notes: Me (Q1; Q3)—median and quartiles (first and third); TD—tardive dyskinesia; CPZeq—chlorpromazine equivalent (calculated according to [46]).

**Table 4:** Results of regression analysis of association between genetic markers and tardive dyskinesia.

Gene	SNP	Estimate	Standard Error	*p*-Value
*GRM3*	rs2237562	0.3884	0.1400	0.0055
*GRM3*	rs1468412	0.3311	0.1400	0.0180
*SLC1A2*	rs1042113	0.3846	0.1434	0.0073
*SLC1A2*	rs10768121	−0.2963	0.1352	0.0284
*SLC1A2*	rs12361171	−0.2963	0.1358	0.0291
*SLC1A3*	rs2229894	−0.3327	0.1401	0.0175

Notes: Additive genetic model was tested using logistic regression adjusting for age and sex. Only nominally significant associations are shown.

**Table 5:** Results of regression analysis of association between haplotypes of *SLC1A2* gene (SNPs rs1042113, rs10768121, and rs12361171) and tardive dyskinesia.

Haplotype	Frequency	OR	95% CI	*p*-Values
TCA	0.3805	1.00 (Ref.)		
CAT	0.2731	1.57	1.15–2.14	0.0048
TAT	0.3311	1.16	0.84–1.60	0.3570
Rare	0.0152	0.74	0.21–2.54	0.6296

Notes: Adjustments were made for age and sex; rare haplotypes with frequency < 0.01 are combined.

## Data Availability

The datasets generated for this study will not be made publicly available, but they are available upon reasonable request to S.A.I. (ivanovaniipz@gmail.com) following the approval of the Board of Directors of the MHRI, in line with the local guidelines and regulations.
